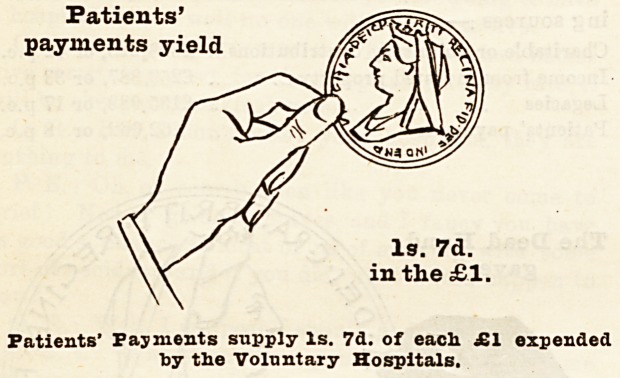# Special Hospital Sunday Supplement

**Published:** 1896-06-13

**Authors:** 


### HOSPITAL SUNDAY MOTTO:—

"He Gives Twice who also Makes his Neighbour Give."—June 14, 1896.

A CORNER IN THE CHILDREN'S WARD.

### Special Hospital Sunday Supplement.

### DEATH THE DESTROYER.

STALKING silently through the great city the destroyer claims his prey. By day and by night, year in year out, oblivious of parties, of politics, of wars and rumours of war, of strikes and of commercial crises, oblivious of all except his own grim office, the man with the scythe gathers in his ghastly harvest, and *in London, this busy hive where all seems so full of life and movement, every six minutes someone dies*. It is only by recalling the melancholy fact that on an average 238 Londoners die every day that one can realise the immensity of sickness and of sorrow that is constantly present all around us. Two hundred and thirty-eight deaths in a single day, and that repeated for every day in the year! Each death breaking up some family or some home, and each death the climax of an illness that may have been dragging on for weeks! Think, then, of the sickness that these deaths mean! It is difficult to form any conception of the amount of illness of one sort and another that exists in London. Sickness in its infinite variety is incapable of enumeration, but deaths can be numbered, and if we think how often we hear and talk of the illness of people within our own immediate circle, and how seldom each circle is invaded by actual death, we may roughly guess at the enormous mass of sickness which is represented by 238 *deaths every day*.

A Thumbnail Sketch, with apologies to Mr. H. H. La Thangue.

### THE DAILY SICK LIST OF LONDONERS.

Those who have gone into such matters statistically reckon that for every case of death in a community there are two persons constantly sick; so that the 86,937 deaths which occurred last year in London represent a daily sick list of no less than 173,874 individuals, all of whom had every day in the year to be looked after by their more healthy brethren. Such numbers are difficult to understand; the mind only takes cognisance of them in a vague and uncertain manner. Let us then place the daily list of sick in London alongside of some other standard of comparison. Take, for example, the British army, in England, in India, and scattered over all our colonies. If we add together all the infantry and cavalry, and all the artillery of every class—horse, field, mountain, and garrison—we do not exceed the daily list of sick in London. Again the sick people in London every day exceed in number the entire population—men, women, and children—of either Brighton, Cardiff, Bolton, Oldham, or Sunderland, and nearly equal that of Leicester. But even the daily sick list gives but a small idea of the vast numbers who are attacked by illness every year. It has been estimated by statisticians that the average length of an illness, *i.e.*, an illness that incapacitates, is 23·2 days, and a simple calculation on this basis gives us the enormous total of 2,608,111 illnesses a year in London.

### THE FACTS OF THE CASE.

All this, however, is mere estimate. The facts are quite as bad as the estimate. Let us turn to facts. Putting on one side the enormous number of patients who are treated by private practitioners, and all those who are lying ill in workhouse infirmaries or are attended by poor law medical officers, let us merely add up in solid figures the numbers actually treated at the voluntary hospitals and the hospitals of the Asylums Board, and then we find ourselves (not as the result of any estimate, but by mere addition) face to face with a fact difficult to realise in its immensity, that *the hospitals alone have during the year* 1894 *treated* 1,790,835 *sick people*. The truth is, that London is permeated on every side with sickness, and that, active and full of life as London seems when looked at from the streets, the amount of illness that is buried away out of sight in courts and alleys, in sick wards, and in the out-patient departments of the hospitals is almost beyond the bounds even of the imagination. Death, doubtless, comes to everyone in the end, but to some much more quickly than to others.

### WHOM DEATH MOST FAVOURS.

The man with a scythe has a preference for infants and old people, and where they predominate there his visits are most frequent. He also loves to see people in overcrowded houses, surrounded by dirt and insanitation, living amid conditions in which the treatment of the sick is an impossibility; for just in those places, such is the irony of fate, sickness comes most often, and just there he gathers his most plentiful harvest. Woe, then, to those who cannot obtain medical aid and nursing, to those who depend for food upon their daily work and, when ill, must starve as well as suffer—woe to them all! for unless some good Samaritan will help them the grim Destroyer will mow them down, and they will die. Who, then, will be the good Samaritan? That is the question of the moment, and the answer is that we must all take our share in the good work by helping the hospitals to help the sick

### THE DISEASES FROM WHICH LONDONERS SUFFER.

IN order to show the proportion borne to one another by the various classes of disease from which the inhabitants of London suffer, we have prepared the following diagrams, which are all drawn to scale, so that the size of the several squares in which the little drawings are contained are exactly proportionate to the number of patients applying to the hospitals in the years 1893 and 1894 for the treatment of the different classes of disease which are named below.

The cases which we have sorted out in this way comprise those treated at the voluntary hospitals and dispensaries of London—including the endowed hospitals, St. Bartholomew's, Guy's, and St. Thomas's—and also at the hospitals of the Metropolitan Asylums Board. They amount altogether to the enormous total of *one million seven hundred and ninety thousand eight hundred and thirty-five patients.* All these were treated during one year, viz., 1894, and to this year only the letterpress refers, although the figures and proportions for 1893 are shown in another set of diagrams also.

**Patients Suffering from Surgical Diseases.**—Of the whole number of patients received by the hospitals *eight hundred and ten thousand seven hundred and three* required surgical treatment. What is meant by "surgical diseases"? They include all accidents, *i.e.*, broken bones, smashed limbs, fractured skulls, and all manner of fractures, displacements, and crushings of sensitive parts or organs. They further include abscesses, ulcerations, cancers, and tumours of all kinds; and, indeed, every injury which accident or pathological process may produce. Surgical diseases include all accidents and all lesions which may be dealt with by hand or instrument. Let anyone who desires to realise what surgical diseases mean for the population of London try to realise this army of 810,703 persons suffering from one or more of the injuries here briefly summarised.

**Patients Suffering from Medical Diseases.**—*Six hundred and twenty-two thousand eight hundred and sixteen* cases received medical treatment. What does the medical profession mean by medical diseases? Diseases which are situated in their entirety or as to their source and origin in one or other of the three great cavities of the body, many of them deep-seated, most of them removed from sight, the diagnosis of their nature and extent is dependent upon the scientific knowledge of the doctor to whose treatment they are committed. They include typhoid, rheumatic, scarlet and other fevers, small-pox, measles, chicken-pox, pneumonia, pleurisy, bronchitis, every kind of heart disease, many forms of brain lesion, diseases of the stomach, bowels, liver, kidney, bladder, pancreas and spleen, most nervous diseases, dyspepsia, constipation, headaches, sleeplessness, and a myriad other ailments, many of them serious, and often resulting in grave danger to life, or at least to the useful existence of mankind. Imagine for a moment considerably more than half a million human beings in London alone attended, free of cost to the patients, by the leading physicians of the day within the buildings of the hospitals of London.

**Patients Treated at Special Hospitals for Children.**—*One hundred and twenty-four thousand two hundred and nine* young people grievously ill sent from homes where they could not be properly treated or even carefully attended to. The fine illustration which is presented with this special number of THE HOSPITAL will bring to the mind of every sympathetic person who studies it what children have to suffer, and how essential it is to the well-being of the race that they should have the skilled medical and surgical care which the hospitals can alone supply to the most grievous of the many cases which arise amongst a large population like that of the metropolis of the British Empire. Surely no words are needed to make the younger residents in this great city determine to give something, even from a limited income, to help suffering children to secure a return to health, and the power of one day shielding themselves against the dangers and risks to life of a vast city like London.

**Patients Suffering from Eye Affections.**—*One hundred and nine thousand four hundred and seven* persons are annually treated by the ophthalmic hospitals of London. It will be seen that, apart from the blind, there were over one hundred thousand people suffering from various forms of disease of the eye which often entailed excruciating pain, and must in many cases have terminated in loss of sight had it not been for the treatment they received at the hospitals. Those who are blessed with sight will surely give something to the hospitals on Hospital Sunday as a thank-offering for their escape from one of the most cruel diseases to which the human frame is liable.

**Diagrams of Diseases.—1893.**

667,028 Surgical Patients.

584,760 Medical Patients.

127,414 Children.

91,324 Eye.

86,622 Women.

46,799 Consumption.

44,312 Throat & Ear.

39,805 Skin.

21,804 Fever.

7,319 Paralysis.

### DISEASES FROM WHICH LONDONERS SUFFER—*continued.*

**Diseases of Women and Motherhood.**—*Seventy-six thousand one hundred and twenty-five* women were treated at the metropolitan voluntary hospitals, the major portion of whom attended the special hospitals for women and the lying-in institutions. Sons of good mothers, and, indeed, all sons must necessarily be moved to pity by realising that, apart altogether from the diseases to which all are liable, woman has to face others which are peculiar to her sex and which entail an immensity of suffering, if they do not render the sufferer permanently disabled from the enjoyment of life and the pursuit of occupations which render life happy and profitable. This group of diseases must excite the sympathies of the most careless, and we make bold to believe that it will stir up many persons to give something this year to the hospitals if they have never yet enjoyed the luxury of investing a handsome sum in so noble a cause.

**Patients Suffering from Consumption.**—*Forty-six thousand five hundred and twenty-two* persons suffering from phthisis or consumption were treated at the consumption hospitals of London during the year. Consumption is the curse of our climate. It spares neither the peer nor the pauper. It attacks the young, the old, and the middle-aged, whilst its ravages defy the highest skill of the greatest physicians of modern times. Who can say with certainty that he may not fall a victim to this dire disease, and who can find it in his heart to deny something towards the cost of alleviating the sufferings of those who have been stricken with this terrible malady?

**Patients Suffering from Diseases of the Ear and Throat.**—*Forty-three thousand four hundred and seventy-two* persons were treated at the special hospitals or special departments devoted to these diseases. The ear, throat, and nose are intimately connected, and large numbers of people require a visit to one of the special hospitals devoted to the ailments included in this section. Residents in a city like London, including both children and adults, are specially liable to these affections, which may involve temporary or permanent impairment of hearing, swallowing, breathing, and even speaking. Consider what it would mean to anyone of us to suffer from one or all of these affections. A little quiet reflection will surely awaken sympathy for those who thus suffer.

**Patients Suffering from Diseases of the Skin.**—*Forty-eight thousand nine hundred and two* patients were treated for skin diseases in London during the year. When it is remembered that many people of both sexes are terrified by the appearance of any affection of the skin, even in these days of advanced science, when immediate alleviation is possible, and permanent relief in the majority of cases assured, little need be said to awaken the sympathy of the reader for the many thousands who annually suffer from ailments of this description. We confidently claim a liberal offering from every inhabitant of London towards the funds needed to rescue those who have to suffer from the extremely common but essentially disagreeable illnesses included in this section.

**Patients Suffering from Fever.**—The number of people who suffered from the various forms of fever during the year amounted to 19,224. Of these a considerable proportion were treated at the hospitals of the Metropolitan Asylums Board. But the term fever includes much besides the class of fevers which are usually removed to these hospitals. All over London measles prevails so largely as to cause three times as many deaths as occur from scarlet fever, and more even than take place from diphtheria, and all these innumerable cases of measles have to be attended in the homes of the people. Moreover, of the diseases notified in 1894 37·1 per cent. of the scarlet fever cases, 67 2 per cent. of the diphtheria cases, and 84·1 per cent. of the typhoid fever cases were not removed to the Asylums Board hospitals. Except a few of the typhoid cases which were taken to the general hospitals, and some of the diphtheria cases which were carried off to the general hospitals in order that operations should be done to prevent impending suffocation by the disease, the great mass of these patients were treated in their own homes. The treatment of fever in its various forms has then fallen in a very much larger degree than many people imagine to the medical officers of the many charitable dispensaries scattered over London.

**Patients Suffering from Paralysis and Diseases of the Nerves.**—*Thirteen thousand six hundred and sixty-four* people were stricken with paralysis and diseases of the nerves, and received treatment at the several hospitals devoted to these maladies. In the morning a man rises from his couch in robust health, and leaves his home to pursue his ordinary avocations. Whilst he is in the counting-house, or the study, or the office, or in the shop following his ordinary business, paralysis seizes him, and he is carried home insensible, helpless, and incapable of uttering a word. No disease is more appalling in its suddenness than paralysis, and it should be matter for thankfulness to Londoners that the hospitals and dispensaries of London were able during the year to give succour to no less than 13,664 cases of paralysis and diseases of the nerves. No one can doubt the association of nervous breakdown with the toil and moil of London life. Let those who are still spared give freely to relieve those who have fallen out of the race, and to keep open the hospitals where immediate treatment can be given to whomsoever may be struck down by those diseases.

**Diagrams of Diseases.—1894.**

810,703 Surgical Patients.

622,816 Medical Patients.

124,209 Children.

109,407 Eye.

76,125 Women.

46,522 Consumption.

43,472 Throat & Ear.

48,902 Skin.

19,224 Fever.

13,664 Paralysis.

### Our Crowded London and its Hospitals.

That he "knows his London well" is the boast of many a man who, if he recognised the responsibility which such knowledge entailed upon him, might hesitate vastly before he spoke so confidently of his knowledge of this city of ours, in which we live so pleasantly when the money-bags are full, and so miserably and meagrely when want of cash prevails. For knowledge involves responsibility, and no one, knowing what is daily going on around, could for shame refuse, or hold his hand, when the hospitals, like poor Oliver, "ask for more." What are the facts? As in literature and in art, so in the more bedraggled modes of earning a livelihood, everyone crowds to be near the centre of employment. The advantages of being on the spot are so apparent that the five million people who form the population of London compete among each other for the dwellings which are situated within reach of work; and thus it has happened that rents have risen and overcrowding has taken place to such an extent that these two features of London life—high rent and crowded dwellings—have come to be the two predominant factors in the misery, sickness, and death of the London poor. We have in this London of ours an enormous multitude of 214,843 persons living in single-room tenements, each with more than two persons to a room, besides all the single people and couples who occupy single rooms. In many cases whole families are thus huddled together, hoping to save in rent what at least may buy them bread. Now what are these people to do when sickness comes unless they go to hospital? This is the question which appeals to those who really know their London well. It is absolutely impossible to treat the sick in these single room tenements, and if civilisation requires that people should, for the due development of the mightiness of this mighty city, live huddled together in this way, civilisation is bound also to supply some sort of accommodation for them when they are overtaken by the sickness which falls to the lot of all. For these, then, the hospitals are an absolute necessity.

This is, however, but a small portion of the truth. A far larger number of people live in tenements of only two rooms than in the single rooms we have just spoken of, and, in fact, in every condition of working London overcrowding is to be reckoned with as the first and most prominent feature in the domestic life of the London workman, the London working woman, and (Heaven help her!) the London working mother.

We feel sure that it will come as a surprise to many to be told that considerably more than one-sixth of the entire population of London live in dwellings which are described by Mr. Shirley Murphy, medical officer of health to the County Council, as "overcrowded tenements"; that is, as defined by the Registrar General, in tenements of four rooms and under having more than two occupants per room. Of larger tenements no note is taken in this enumeration, however much overcrowded they may be, nor of small ones so long as they do not contain more than two persons to each room. Now what can be done for the proper treatment of illness when there are only from one to four rooms, and already everyone of them, including the living room, has its more than two sleepers to accommodate? Many people profess to think that London is overdone with hospitals. To these we point out the fact that the necessity for large hospita accommodation in London arises, to a great extent, from the absence of home accommodation in which those suffering from sickness can obtain decent attendance, never to mention proper treatment for their maladies. *Seven hundred and twenty-nine thousand eight hundred and sixty-five persons live in tenements, which, according to the medical officer of health, are too small for them even when they are well!* Who can deny the necessity for hospitals in the face of such a fact as this?

If sickness falls as a heavy blow upon the wealthy and the highly placed, even when every means is at their disposal to ward it off and lessen its discomforts, how much more serious a matter is it to those who, born amid bad surroundings, and reared on adulterated food, have to live amidst the degrading influences of overcrowding and to labour for every scrap of food they eat? Such people have sickness almost always with them, and so far as such lives are a product of our day so should our day provide help and comfort when sickness falls upon them, and this can only be done by giving liberally to the hospitals which have been raised for the purpose on every side.

To measure the relation of health to roominess of dwelling, and of sickness to overcrowding, Mr. Shirley Murphy, in the report above alluded to, has arranged in series the different districts in accordance with the percentage of overcrowded dwellings in each, with the result that he finds that the death-rate steadily increases in proportion to the overcrowding. Just where the home is only capable of providing the merest shelter, just where there are absolutely no appliances for the treatment of illness, no room for a nurse, no room in which to keep comforts for times of sickness, even if there were money to buy them, just in those places where every corner is so occupied that the doctor on his rounds has to keep his hat on his head because there is no other place where he can put it; there sickness occurs with far greater frequency, and the hand of death falls with greater heaviness than in more comfortable homes, where, at least, if sickness comes, some countervailing comfort can come also; and, if death appears, at least some preparation can be made. That the sick from these overcrowded quarters should be removed to hospital is then one of the necessary consequences of that overcrowded civilisation of which this century appears to be so proud, and those who know their London must accept the obvious corollary of their knowledge. To London, as it stands at present, the hospitals are an absolute necessity, and the support of the hospitals is the first duty of every dweller in this great city.

### Embarrassing Popularity.

The demand for this Supplement before publication this year was greater than we could supply, although 25,000 copies were printed. We ask the co-operation of everyone into whose hands a copy may fall to pass it on and to send at least £1 for the hospitals to the Right Hon. Sir Walter Wilkin, the Lord Mayor, at the Mansion House, London, E.C.

### The Sanctuary of the Sick.

We must offer our apologies to Mr. L. J. Pott for having enlisted the subject of his famous picture in the cause of the hospitals.

**Disease repelled by Medicine and Nursing, with the aid of the Church.**

The Jews of old were a justice-loving race. An eye for an eye, a tooth for a tooth, yea, and a life for a life was the creed in which they were brought up. But amid all the stern severity of their laws there was one green oasis of human sympathy and pity, for cities of refuge were appointed to which the innocent manslayer might fly, and in which he might be safe from his pursuer. In Christian times, too, all through the troublous Middle Ages, when often neither man's life nor woman's virtue was safe from the toils of the oppressor, God's churches offered sanctuary, and even sinners might feel safe if they sought protection from the priests and cast themselves at the foot of the altar. The right of sanctuary was a precious one, a powerful protection from the evils of the times. The modern sanctuary is a different affair. New times, new manners! We do not now dwell in fear of murder and rapine, but death none the less dogs the footsteps of the people although in different form, and when we seek sanctuary nowadays it is against disease, the vanguard of the advancing foe.

With the lessening power of the individual oppressor has grown up the harsh tyranny of a crowded civilisation. As the direct outcome of the vices of mankind, and as the product of the modes of life which are forced upon the people by our civilisation, our artificial foods, our crowded houses, our abnormal hours, our monotonous toil, our ever advancing division of labour, diseases arise which are not only injurious to those on whom they fall, but tend to be transmitted to future generations, and thus if man would escape the destroyer he must seek sanctuary now even as in times gone by, but this time at the hospital gate instead of at the altar steps.

The hospital is the modern sanctuary, where the sick take refuge, and the God of Healing with his ministering angels in the guise of nurses keeps at bay the multitude of diseases which assail the dwellers in great cities. Just as the unflinching sternness of the Jewish law had to be tempered by the mercy offered in the cities of refuge, and as the wild oppression of the Middle Ages was only made bearable by virtue of the safe sanctuary offered at the altar, so in these days the artificial and abnormal life led by the masses in our great cities is only rendered tolerable by the sanctuary against disease offered by our great voluntary hospitals in the name of charity. The crowds of people flocking day by day to the hospitals, seeking there that safety which they cannot find elsewhere, is, to some extent, a measure of the necessity of these benecent institutions. Reference to the figures appended to this Hospital Sunday Supplement will show how great is the work that is being done, and how great the need for help in doing it. That in a single year 1,790,835 cases should apply for treatment at our hospitals is an enormous fact. It is distressing, but it is true; and it is to a large extent the outcome of the mode of life led by the masses in our great metropolis. Disease is bad anywhere, but nowhere is it so terrible as in the overcrowded dwellings which, as is shown in another article, are so common in London. Some help is absolutely necessary, and it is clear that to offer freely and ungrudgingly a safe sanctuary against disease is the least that can be done by a great city which, while growing vast and powerful, does so at the expense of much degeneration and unavoidable disease among its people.

### RATES, CHARITY, AND COMMON SENSE.

A DIALOGUE OF THE DAY.

INDIGNANT AND MUCH OPPRESSED RATEPAYER: Why should I subscribe to these hospitals? What have they done to help me, I should like to know?

CHARITABLY DISPOSED PERSON: Well, you know charity is a good thing to those that give as well as to those who receive, and I always notice that free givers are happy men, at any rate, they seem happier than those who are close-fisted.

POLITICAL ECONOMIST: That's all very well, but I think all these sort of things should come upon the rates.

OPPRESSED RATEPAYER: Oh, bother that; we've got rates enough as it is, what with School Board rates and Asylum Board rates. If you are going to have a hospital rate as well no one will be able to live.

COMMON SENSE INDIVIDUAL: Then you had better give something down for the hospitals; at any rate, it will be cheaper than fresh rates.

O. R.: But I don't want your hospitals, they are nothing to me.

P. E.: Oh, of course, men like you never come to grief! Now I go on averages, and I fancy you have as good a chance as most of us of meeting with some sort of accident, and if you did, what would happen to you?

O. R.: Well, I suppose I should go home.

C. S. I.: Why, man alive, you are miles from home half your time. Accidents can't be arranged to suit your own convenience, to be delivered to order just at your door-step.

O. R.: Well, anyhow, I could go to an hotel.

C. S. I.: And a nice little bill you would have to pay! Why I knew a fellow who was knocked over by a hansom. It was done before you could wink; over he went, and what would he have done without a hospital? Hotel indeed! Why before he came round to say who he was or where he would like to go he was between the sheets with a couple of jolly nice nurses looking after him and a doctor putting his leg right, and there he had to lie for nigh upon a month before he could be moved. A nice little bill he would have had to pay for himself and his nurses, and all the odds and ends he wanted all that time! No, my dear fellow, it can't be done. You must average things. You are as likely to be smashed as anyone else, and you must arrange that if you are smashed there shall be somewhere for you to go.

C. D. P.: But don't you think that if our friend here was "smashed," as you call it, someone would be found to take him in?

C. S. I.: No, not a bit of it. We've got used to having hospitals, and we don't do our charity that way nowadays. System, my dear fellow—system is everything. If we are little people we give a guinea; if we are big people we give a hundred guineas to a neighbouring hospital; or if we cannot be bothered to find out which hospital to give to we send it to the Hospital Sunday Fund, which takes all responsibility in the matter off our hands. But in any way we are clear of the affair, and if a man falls in a fit, or an old woman is run over, we do not feel called upon to take them in, and tuck them up in our best bed-rooms, and tend them till they are sound again. No. It may seem a drag having to subscribe to hospitals, but at least it saves us from what would be a most disagreeable tax on every householder, liable to fall on him at any moment if no hospitals existed.

O. R.: That's a tax I wouldn't pay.

C. D. P.: Oh, I am sure when it came to the point you would be very glad to do all that was kind and proper.

O. R.: Not I!

P. E.: Oh! but you would have to! It is difficult to imagine a city like London without hospitals; but certainly, if such a thing could be, you would quickly find that public opinion would have a voice in the matter, and that, whether you liked it or not, if an accident happened in front of your house you would have to take it in. You wouldn't let the wretch die in the street, would you?

O. R.: Well, no, not die, of course.

C. D. P.: No, I know you wouldn't.

C. S. I.: Well, then, stump up for the hospitals for doing for you what you would have had to do for yourself if they had not existed. Besides, there are other reasons. When your boy was ill did you not have an operation done?

O. R.: Yes; but I paid for it, and no small sum either.

C. S. I.: Oh, you think so, do you? You paid the doctor for his skill, but where did he get his skill? It seems to me that he got his skill from the hospital, and you do not seem to have paid the hospital yet for the recovery of that son of yours.

O. R.: But that's nonsense.

C. S. I.: Not a bit! Do you think that if your doctor had spent his time sitting in his study waiting for patients he would ever have been able to do that operation in the way he did do it? or even if he had studied all the books in creation? No, my friend, you owe that boy to the hospital; and, for the matter of that, it is not only in big operations that hospitals come in, but in every little detail in medical practice. It is the training in the hospitals that has made doctors powerful to cure, and it is the knowledge gained in hospitals which has led even to the lessened prevalence of disease which is so marked a feature of modern times. Did I not hear you say that you had got some stuff lately that put those headaches of yours right in a jiffy that used to lay you up for a couple of days?

O. R.: Oh, yes; I'm all right now.

C. S. I.: Glad to hear it! But you owe that to the hospitals; and lots of other things—the gas you have when you have a tooth out, the chloroform by which your wife got her last confinement over without knowing anything about it, and all sorts of things besides. Don't tell me that you can live in this part of the century and not share in the benefits of the hospitals; and if you get the benefits, even if you do not use the hospitals, you ought to pay for the latter.

O. R.: Well, here's a couple of sovereigns for the Hospital Sunday Fund.

### A Word to Living Londoners.

### Ways and Means.

In the course of an article in last year's Supplement we pointed out that if the 1s. 6d. in the pound derived from patients' payments be credited to the living, *i.e.*, to the present inhabitants of London, then the sum yielded to the hospitals from the living in 1893 amounted to only 9s. in the pound, as compared with the contribution of 10s. 6d. in the pound from former friends of the hospitals now dead, who cannot in any sense benefit by the existence of our voluntary institutions. We went on to urge the Press to spread these facts widely, so that the living might arouse themselves and increase their contributions on Hospital Sunday so as to make the living hand give at least 10s. 6d. in every sovereign expended upon the care of the sick to-day. We rejoice to notice that the returns for the year 1894 show that, including St. Bartholomew's and St. Thomas's Hospitals, the living gave 8s. 5d. in the pound, or 11d. more than in the previous year; and, if we add to this sum the 1s. 7d. in the pound derived from patients' payments, it brings the contributions from the living up to 10s. in the pound.

The Living, i.e., the present inhabitants. only give 8s. 5d. of each £1 expended by the Voluntary Hospitals.

This result is encouraging, and we venture to hope that our contemporaries will again consent to make the facts known this year in connection with Hospital Sunday, and that in the result another shilling in the pound may be forthcoming from the present residents in London, when the voluntary hospitals will at last be placed upon a sound financial basis. This result will certainly be achieved if the laudable efforts of the present Lord Mayor, Sir Walter Wilkin, to secure the active exertions of the clergy from the pulpits on June 14th, 1896, prove in the main successful. The laity did their part last year by sending £20,000 as a free gift to the hospitals through the Sunday Fund, and as Dr. Rigg has pointed out, this spontaneous exhibition of interest on the part of the laity should stimulate the clergy of all the churches to urgently plead the cause of the hospitals from the pulpits on Hospital Sunday. If they do this there can be no doubt that the extra shilling in the pound will be subscribed, and that the larger portion of it will be given through the contributing congregations, a result which would confer honour upon everyone who contributes to bring it about.

### The Cost of the Work Done.

In the year 1894 the total expenditure of the London voluntary hospitals, including St. Bartholomew's, Guy's, and St. Thomas's, and the metropolitan dispensaries, was £786,976. In other words, it required upwards of three-quarters of a million of money to defray the cost of providing adequate hospital treatment for the patients, who numbered about one million and three-quarters (1,760,000). We have excluded from this calculation 19,224 fever cases treated at the hospitals of the Metropolitan Asylums Board, and the cost of such treatment. The total income of the London voluntary hospitals and dispensaries during 1894 was £785,663, which was derived from the following sources:—

| | | |
|---|---|---|
| Charitable or voluntary contributions... | £328,225, | or 42 p.c. |
| Income from invested property ... ... | £259,387, | or 33 p.c. |
| Legacies ... ... ... ... ... | £135,989, | or 17 p.c. |
| Patients' payments ... ... ... | £62,062, | or 8 p.c. |

The Dead Hand gives 10s. out of every £1 expended by the Hospitals.

In the above figures the income and expenditure of St. Bartholomew's and St. Thomas's Hospitals, being £116,894 and £111,195 respectively, has been confined to that portion of their revenue and expenditure which was applicable to hospital purposes.

### How the Money is Provided.

Let us now consider where the money came from to pay the cost of the hospital relief given to the inhabitants of London by these voluntary institutions. In order to bring home the facts to the meanest comprehension, we have prepared diagrams each representing a hand and a coin, which have been drawn to scale, and show exactly the proportion of every sovereign which was contributed by the living, who received all the benefits, and by deceased benefactors, many of whom took an active part in the management of hospitals during their lifetime, and whose benefactions have enabled them to meet the ever increasing needs of upwards of four millions of people. With a view to clearness and ready comprehension, the diagrams have been drawn so as to represent the proportion given of every sovereign expended in 1894 by (*a*) the living (*b*), the dead, and (*c*) the patients themselves. Of every sovereign expended **10s.**, or *one-half, is derived from legacies and the interest upon gifts* of deceased benefactors which have been invested in approved securities; **8s. 5d.** out of every sovereign has been *given in charity by* the present inhabitants of London, that is, *the living, for whose benefit the hospitals exist;* and **1s. 7d.** out of every sovereign has been *contributed by the patients treated in the hospitals.*

In 1893 the annual deficiency amounted to £23,984, which represented about 6d. in the pound. In 1894 this deficiency has been considerably reduced, but, having regard to the increased demand upon the hospitals, and the growth in the population of London, an additional shilling in the pound is required from the present inhabitants to place the voluntary hospitals once and for all in so strong a position financially that the plea for rate support will be finally rejected, as it ought to be rejected, in the interests of our common humanity.

**Patients' Payments supply 1s. 7d. of each £1 expended by the Voluntary Hospitals.**

We may add that *the black hand* and the coin held by it represent the dead hand, *i.e.*, the contributions from those now dead, *the white hand* represents the charitable contributions of the living, and the smallest coin the amount derived from patients' payments.

THE MEANING OF THE DIAGRAMS.

We desire to direct the attention of all classes in London to the foregoing figures. They show that, whereas the people of London resort to the voluntary hospitals in greater numbers than the population of any other city in the United Kingdom, they still fail to recognise their full duty to the hospitals and to show their sense of the benefits they have received from these institutions. Every provincial city of importance takes the deepest pride in its hospitals and provides them with adequate funds. It follows that in provincial cities, instead of the hospitals being starved and impoverished for want of liberal support at the hands of the living, who are continually demanding and always receiving increasing benefits from these institutions, all classes of the population combine to provide the necessary funds in adequate proportions. In London almost the exact opposite is the case. The living show their appreciation of the hospitals by demanding greater value in relief every year at the hands of the hospitals, whilst they contribute in payment for the benefits they receive about one-half of its total cost. Is there one intelligent citizen of London who will be content to find that the population of this vast city in the year 1894 contributed barely more than two-fifths of the sum which was expended in affording hospital relief to the citizens as a whole?

Had it not been for the contributions of the dead hand, which amount at the present time to 10s. out of every pound, more than half the hospitals must have been closed for want of funds. We appeal to the people of London to exhibit more self-respect and a determination to henceforth follow the noble example set them in this matter by their forebears and sires.

But this is not the whole case, for if the 1s. 7d. in the pound derived from patients' payments be credited to the living, *i.e.*, to the present inhabitants of London, then the sum yielded only amounts to 10s. in the pound, as compared with a contribution of 10s. in the pound from those now deceased who cannot in any sense benefit by the existence of our voluntary hospitals.

We hope that the Press will make these facts widely known, and that the living will arouse themselves sufficiently and so increase their contributions on Hospital Sunday this year that the charitable contributions, combined with patients' payments, will at least amount to 11s. in every sovereign. Although it is true that the total deficiency of all the London hospitals in 1894 represents less than sixpence in the pound, still there are hospitals like Guy's, Charing Cross and King's College which are urgently in need of large sums to defray current expenses, whilst the district of South-east London, containing 1½ millions of the population, has no adequate hospital accommodation at all, a fact which is making itself felt with increasing urgency at the present time. *An extra one shilling in the pound from the living would put all the existing hospitals in funds.*

### THE METROPOLITAN HOSPITAL SUNDAY FUND.

IN the course of their report for 1895 the council state:—

"Amongst the preachers who materially contributed to the splendid results of 1895, mention must be made of the special sermons preached by the Archbishop of Canterbury, the Chief Rabbi, the Dean of Westminster, the Dean of Canterbury, the Archdeacon of London, Canons Ainger, Fleming, Wilberforce, and Keatinge, the Rev. C. J. Ridgeway, and many others who united with the Press to secure the £60,000.

"The editors of the metropolitan press enthusiastically co-operated, and Arthur Walter, Esq., of the *Times*, for the first time in its history, consented to insert and give prominence to the wood-cuts which they re-published from the Special Supplement of THE HOSPITAL, nearly 60,000 copies of which were circulated at the cost of a few friends of the editor, and £18,000 was sent in contributions to the Lord Mayor as the result of THE HOSPITAL appeal. The *Lancet* issued once more a Special Supplement which was widely circulated, and the editors of the *Times, Standard, Daily News, Daily Chronicle, Morning Post, Daily Graphic, Morning Advertiser*, the *Pall Mall, St. James's*, and *Westminster Gazettes*, the *Globe*, and the Sunday and weekly papers all rendered willing assistance, with results so fruitful that the council hope the Press may be encouraged to continue similar efforts year by year, and so to provide that the Metropolitan Hospital Sunday Fund shall never again yield less than £50,000 for the metropolitan medical charities.

"*The working expenses are only* 2·690 *per cent. of the gross receipts.*"

### Hospital Sunday in London, 14th June, 1896.

### THE WORK DONE BY THE HOSPITALS AND MEDICAL CHARITIES IN 1895.

### NEWINGTON AND SOUTH DISTRICT.

Comprising Battersea, Wandsworth, Tooting, Balham, Streatham, Brixton, Lambeth, Newington, Southwark, Bermondsey, Camberwell, Greenwich, Deptford, Lewisham, Blackheath, Woolwich, &c.

| No. of Beds. | No. of Beds Daily Occupied. | HOSPITALS. | In-patients. | Out-patients. | Total Expenditure. | Income. Charitable. | Income. Proprietary. | Income. Patients' Payments. | Total Income. | Legacies not included in previous column. |
|---|---|---|---|---|---|---|---|---|---|---|
| | | | | | £ | £ | £ | £ | £ | £ |
| 621 | 416 | Guy's ... ... ... ... ... | 5,920 | 69,806 | 43,367 | 5,567 | 26,755 | 4,894 | 37,216 | ... |
| 23 | 19 | Miller ... ... ... ... ... | 291 | 11,290 | 3,274 | 2,142 | 374 | ... | 2,516 | 59 |
| 253 | 197 | Seamen's ... ... ... ... | 2,375 | 15,918 | 15,352 | 8,655 | 4,030 | 161 | 12,846 | 705 |
| 66 | 60 | Evelina, for Children ... | 725 | 8,855 | 6,503 | 5,350 | 546 | 90 | 5,986 | 550 |
| 57 | 47 | Home for Sick Children ... ... | 248 | 1,584 | 2,101 | 1,196 | 451 | 332 | 1,979 | 480 |
| 24 | 21 | General Lying-in ... ... ... | 535 | 1,782 | 3,373 | 760 | 2,531 | ... | 3,291 | ... |
| 36 | 14 | Clapham Maternity and Dispensary | 318 | 276 | 1,528 | 323 | 500 | 640 | 1,463 | ... |
| 52 | 43 | Royal, for Children and Women ... | 486 | 8,033 | 4,345 | 2,996 | 1,071 | 259 | 4,326 | ... |
| 40 | 20 | Royal Eye ... ... ... ... | 401 | 14,052 | 3,334 | 1,955 | 84 | 318 | 2,357 | ... |
| 10 | 1 | Hospital for Diseases of the Skin ... | 14 | 4,995 | 1,110 | 219 | 152 | 560 | 931 | ... |
| 352 | 193 | Metropolitan Convalescent ... ... | 2,944 | ... | 6,275 | 6,232 | 630 | 104 | 6,966 | 320 |
| 10 | 5 | Phillips' Memorial Homœopathic ... | 82 | 500 | 557 | 373 | ... | 198 | 571 | ... |
| 10 | 8 | Eltham Cottage ... ... ... | 80 | ... | 484 | 376 | 9 | 113 | 498 | ... |
| 18 | 12 | Beckenham Cottage ... ... ... | 108 | ... | 620 | 552 | 43 | 90 | 685 | ... |
| 20 | 10 | Blackheath Cottage ... ... ... | 163 | 921 | 721 | 793 | 20 | 105 | 918 | ... |
| 20 | 14 | Bromley Cottage ... ... ... | 218 | ... | 1,233 | 774 | 73 | 129 | 976 | 45 |
| 14 | 9 | Chislehurst, &c., Cottage ... ... | 123 | ... | 730 | 463 | 11 | 116 | 590 | ... |
| 12 | 6 | Sidcup Cottage ... ... ... | 94 | 40 | 427 | 353 | ... | 84 | 437 | 100 |
| 1,638 | 1,095 | | 15,125 | 138,052 | 95,344 | 39,079 | 37,280 | 8,193 | 84,552 | 2,259 |
| | | DISPENSARIES. | | | | | | | | |
| ... | ... | Battersea Provident ... ... ... | ... | 18,025 | 2,786 | 116 | 59 | 2,678 | 2,853 | ... |
| ... | ... | Brixton, &c. ... ... ... ... | ... | 4,996 | 723 | 593 | 112 | ... | 705 | ... |
| ... | ... | Camberwell Provident ... ... | ... | 11,695 | 2,012 | 1,812 | 113 | 1,436 | 3,361 | 50 |
| ... | ... | Clapham ... ... ... ... | ... | 1,364 | 326 | 252 | ... | 95 | 347 | 90 |
| ... | ... | Deptford Medical Mission ... ... | ... | 3,372 | 477 | 374 | 49 | 62 | 485 | ... |
| ... | ... | East Dulwich Provident ... ... | ... | 3,927 | 626 | 86 | ... | 545 | 631 | ... |
| ... | ... | Forest Hill ... ... ... ... | ... | 2,189 | 687 | 282 | 4 | 435 | 721 | ... |
| ... | ... | Gipsy Hill, &c. ... ... ... | ... | 516 | 120 | 41 | ... | 73 | 114 | ... |
| ... | ... | Royal South London... ... ... | ... | 4,357 | 693 | 598 | 112 | ... | 710 | ... |
| ... | ... | South Lambeth, &c. ... ... ... | ... | 2,206 | 553 | 344 | 14 | 181 | 539 | ... |
| ... | ... | Walworth Provident ... ... | ... | 750 | 231 | 16 | 35 | 106 | 157 | ... |
| ... | ... | Wandsworth Common ... ... | ... | 835 | 212 | 56 | ... | 156 | 212 | ... |
| 1,638 | 1,095 | | 15,125 | 192,284 | 104,780 | 43,649 | 37,778 | 13,960 | 95,387 | 2,399 |

### CITY AND EAST CENTRAL DISTRICT.

Comprising the City, St. Luke's, Shoreditch, Finsbury, and Clerkenwell.

| No. of Beds. | No. of Beds Daily Occupied. | HOSPITALS. | In-patients. | Out-patients. | Total Expenditure. | Income. Charitable. | Income. Proprietary. | Income. Patients' Payments. | Total Income. | Legacies not included in previous column. |
|---|---|---|---|---|---|---|---|---|---|---|
| | | | | | £ | £ | £ | £ | £ | £ |
| 114 | 70 | Metropolitan ... ... ... ... | 971 | 21,340 | 10,903 | 6,761 | 451 | 1,419 | 8,631 | 772 |
| 160 | 112 | Royal Free ... ... ... ... | 1,802 | 31,893 | 10,896 | 4,101 | 1,102 | ... | 5,203 | 3,921 |
| 80 | 53 | Royal, for Diseases of the Chest ... | 701 | 6,577 | 8,042 | 5,905 | 114 | ... | 6,019 | 3,329 |
| 57 | 47 | North-Eastern, for Children ... | 687 | 15,519 | 5,803 | 4,703 | 585 | 697 | 5,985 | 2,265 |
| 36 | 21 | City of London Lying-in ... ... | 490 | 1,585 | 3,716 | 604 | 3,433 | ... | 4,037 | ... |
| 45 | ... | St. Mark's, for Fistula ... | ... | 614 | 1,062 | 823 | 908 | ... | 1,731 | 956 |
| 100 | 90 | Royal London Ophthalmic... ... | 2,184 | 26,290 | 7,118 | 3,103 | 965 | ... | 4,068 | 637 |
| 53 | 30 | City Orthopædic ... ... ... | 200 | 2,220 | 1,451 | 1,519 | 30 | ... | 1,549 | ... |
| 17 | 9 | Central London Throat and Ear ... | 257 | 6,731 | 2,064 | 612 | 174 | 1,294 | 2,080 | ... |
| 662 | 432 | | 7,292 | 112,769 | 51,058 | 28,131 | 7,762 | 3,410 | 39,303 | 11,880 |
| | | DISPENSARIES. | | | | | | | | |
| ... | ... | City ... ... ... ... ... | ... | 4,920 | 1,272 | 1,045 | 156 | ... | 1,201 | ... |
| ... | ... | City of London and East London ... | ... | 12,329 | 1,161 | 143 | 39 | 1,125 | 1,307 | ... |
| ... | ... | Farringdon General ... ... ... | ... | 5,335 | 632 | 297 | ... | 292 | 589 | ... |
| ... | ... | Finsbury ... ... ... ... | ... | 11,488 | 843 | 407 | 147 | 270 | 824 | ... |
| ... | ... | Metropolitan ... ... ... ... | ... | 7,602 | 920 | 421 | 117 | 325 | 863 | ... |
| ... | ... | Royal General ... ... ... ... | ... | 4,083 | 972 | 720 | 233 | 101 | 1,054 | ... |
| 662 | 432 | | 7,292 | 158,526 | 56,858 | 31,164 | 8,454 | 5,523 | 45,141 | 11,880 |

### ST. MARYLEBONE AND WEST CENTRAL DISTRICT.

Comprising St. Marylebone, St. John's Wood, Bloomsbury, Holborn, &c.

| No. of Beds. | No. of Beds Daily Occupied. | Hospitals. | In-patients. | Out-patients. | Total Expenditure. | Income. Charitable. | Income. Proprietary. | Income. Patients' Payments. | Total Income. | Legacies not included in previous column. |
|---|---|---|---|---|---|---|---|---|---|---|
| | | | | | £ | £ | £ | £ | £ | £ |
| 70 | 47 | French ... ... ... ... ... ... | 755 | 4,184 | 4,367 | 4,082 | 41 | ... | 4,123 | 1,320 |
| 20 | 12 | Italian ... ... ... ... ... ... | 237 | 4,808 | 845 | 716 | 91 | ... | 807 | ... |
| 70 | 50 | London Homœopathic ... ... | 565 | 11,158 | 6,364 | 2,007 | 2,776 | 478 | 5,261 | 300 |
| 51 | 40 | SS. John and Elizabeth ... .. | 94 | ... | 1,641 | 710 | 845 | ... | 1,555 | 664 |
| 321 | 290 | The Middlesex ... ... ... | 3,404 | 42,922 | 38,012 | 11,133 | 12,499 | ... | 23,632 | 12,358 |
| 68 | 62 | Alexandra, for Children ... ... | 159 | 249 | 2,581 | 1,981 | 142 | 418 | 2,541 | 100 |
| 30 | 29 | Hospital for Incurable Children ... | 37 | ... | 1,176 | 649 | 37 | 434 | 1,120 | ... |
| 238 | 155 | Hospital for Sick Children ... ... | 1,861 | 21,808 | 15,463 | 8,636 | 3,779 | ... | 12,435 | 5,732 |
| 16 | 9 | British Lying-In ... ... ... | 187 | 372 | 1,793 | 517 | 1,157 | ... | 1,674 | ... |
| 55 | 43 | Queen Charlotte's Lying-In ... | 1,124 | 1,370 | 4,991 | 4,147 | 1,439 | 16 | 5,602 | 1,106 |
| 42 | 35 | New Hospital for Women ... ... | 534 | 12,030 | 3,980 | 2,225 | 204 | 1,231 | 3,660 | ... |
| 53 | 44 | Samaritan Free ... ... ... | 568 | 9,205 | 6,638 | 3,912 | 229 | ... | 4,141 | 781 |
| 180 | 168 | National, for the Paralysed, &c. ... | 952 | 5,065 | 13,460 | 5,716 | 1,593 | 1,799 | 9,108 | 14,074 |
| 25 | 19 | Hospital for Epilepsy, &c. ... ... | 90 | 711 | 1,853 | 853 | 53 | 632 | 1,538 | 88 |
| 50 | 30 | West End, for Epilepsy, &c. ... | 217 | 2,669 | 2,508 | 2,111 | 41 | 474 | 2,626 | ... |
| 13 | 5 | Central London Ophthalmic ... | 170 | 9,521 | 920 | 352 | 78 | 456 | 886 | ... |
| 25 | 9 | Western Ophthalmic... ... ... | 220 | 6,274 | 859 | 645 | 135 | ... | 780 | 290 |
| 60 | 57 | National Orthopædic ... ... | 216 | 748 | 2,264 | 824 | ... | 1,292 | 2,116 | ... |
| 25 | 15 | Establishment for Gentlewomen ... | 123 | ... | 2,286 | 780 | 131 | 916 | 1,827 | 200 |
| ... | ... | National Dental ... ... ... | ... | 34,669 | 7,824 | 1,268 | ... | 250 | 1.518 | ... |
| 1,412 | 1,119 | | 11,513 | 167,763 | 119,825 | 53,264 | 25,290 | 8,396 | 86,950 | 37,013 |
| | | Dispensaries. | | | | | | | | |
| ... | ... | Bloomsbury Provident ... ... | ... | 428 | 251 | 17 | ... | 189 | 206 | ... |
| ... | ... | London Medical Mission ... ... | ... | 2,123 | 1,119 | 718 | 80 | 139 | 937 | 325 |
| ... | ... | Portland Town ... ... ... | ... | 500 | 153 | 144 | 5 | 15 | 164 | ... |
| ... | ... | Portobello Road ... ... ... | ... | 149 | 80 | 34 | 4 | 30 | 68 | ... |
| ... | ... | St. John's Wood Provident ... | ... | 4,798 | 695 | 277 | 35 | 302 | 614 | 100 |
| ... | ... | St. Marylebone General ... ... | ... | 5,263 | 991 | 456 | 127 | 343 | 926 | ... |
| ... | — | Western General ... ... ... | ... | 18,032 | 1,357 | 1,316 | 31 | 11 | 1,358 | 25 |
| 1,412 | 1,119 | | 11,513 | 199,056 | 124,471 | 56,226 | 25,572 | 9,425 | 91,223 | 37,463 |

### KENSINGTON AND WEST DISTRICT.

Comprising Kensington, Paddington, Bayswater, Kilburn, Chelsea, Brompton, Fulham, Hammersmith, Chiswick, Brentford, Acton, Ealing, &c.

| No. of Beds. | No. of Beds Daily Occupied. | Hospitals. | In-patients. | Out-patients. | Total Expenditure. | Income. Charitable. | Income. Proprietary. | Income. Patients' Payments. | Total Income. | Legacies not included in previous column. |
|---|---|---|---|---|---|---|---|---|---|---|
| | | | | | £ | £ | £ | £ | £ | £ |
| 351 | 280 | St. George's ... ... ... ... | 3,703 | 28,819 | 42,450 | 10,337 | 15,407 | ... | 25,744 | 33,319 |
| 281 | 246 | St. Mary's ... ... ... ... | 3,968 | 38,602 | 22,229 | 17,615 | 2,122 | ... | 19,737 | 5,390 |
| 101 | 93 | West London ... ... ... ... | 1,647 | 32,410 | 6,700 | 4,877 | 201 | ... | 5,078 | 1,850 |
| 321 | 276 | Hospital for Consumption ... ... | 1,543 | 13,750 | 28,953 | 12,623 | 8,339 | ... | 20,962 | 3,633 |
| 24 | 20 | Belgrave, for Children ... ... | 270 | 3,905 | 1,846 | 1,356 | 96 | ... | 1,452 | 500 |
| 50 | 49 | Cheyne for Sick & Incurable Children | 67 | ... | 2,610 | 1,825 | 250 | 503 | 2,578 | ... |
| 30 | 21 | Paddington Green, for Children ... | 298 | 11,687 | 2,179 | 5,948 | 63 | 200 | 6,211 | 145 |
| 120 | 115 | Victoria, for Children ... ... | 1,690 | 18,770 | 8,308 | 5,779 | 370 | 484 | 6,633 | 1,220 |
| 74 | 45 | Chelsea, for Women ... ... | 496 | 2,622 | 5,758 | 5,410 | 112 | 599 | 6,121 | 395 |
| 105 | 81 | Cancer ... ... ... ... ... | 805 | 1,428 | 11,754 | 7,192 | 2,467 | ... | 9,659 | 19,718 |
| 135 | 95 | Female Lock ... ... ... ... | 709 | ... | 3,886 | 1,785 | ... | 1,956 | 3,741 | 1,560 |
| 22 | 21 | Male Lock ... ... ... ... | 391 | 4,953 | 1,903 | 100 | ... | 1,787 | 1,887 | ... |
| 16 | 9 | Epsom and Ewell Cottage ... ... | 111 | ... | 870 | 721 | 4 | 198 | 923 | ... |
| 18 | 15 | Reigate and Redhill Cottage ... | 204 | ... | 792 | 518 | 51 | 99 | 668 | 45 |
| 11 | 6 | Wimbledon Cottage ... ... ... | 113 | ... | 458 | 403 | 5 | 64 | 472 | ... |
| 13 | 7 | Hounslow Cottage ... ... ... | 80 | 986 | 439 | 363 | 182 | 52 | 597 | ... |
| 1,672 | 1,379 | | 16,095 | 157,932 | 141,135 | 76,852 | 29,669 | 5,942 | 112,463 | 67,775 |
| | | Dispensaries. | | | | | | | | |
| ... | ... | Brompton Provident ... ... | ... | 1,437 | 508 | 159 | 82 | 264 | 505 | ... |
| ... | ... | Chelsea, &c. ... ... ... ... | ... | 5,206 | 762 | 495 | 184 | ... | 679 | ... |
| ... | ... | Chelsea Provident ... ... ... | ... | 418 | 256 | 32 | ... | 178 | 210 | ... |
| ... | ... | Kensal Town Provident ... ... | ... | 510 | 331 | 32 | 53 | 260 | 345 | ... |
| ... | ... | Kensington ... ... ... ... | ... | 4,178 | 673 | 663 | 49 | ... | 712 | ... |
| ... | ... | Kilburn, Maida Vale ... ... | ... | 3,303 | 511 | 434 | 52 | ... | 486 | ... |
| ... | ... | Kilburn Provident ... ... ... | ... | 5,018 | 1,083 | 77 | 12 | 995 | 1,084 | ... |
| ... | ... | Notting Hill Provident ... ... | ... | 184 | 167 | 85 | — | 50 | 135 | ... |
| ... | ... | Paddington Provident ... ... | ... | 3,911 | 615 | 189 | 25 | 366 | 580 | ... |
| ... | ... | Pimlico Provident ... ... ... | ... | 2,305 | 530 | 25 | ... | 592 | 617 | ... |
| ... | ... | Royal Pimlico Provident ... ... | ... | 7,240 | 958 | 348 | 30 | 540 | 918 | ... |
| ... | ... | Westbourne Provident ... ... | ... | 992 | 396 | 56 | 44 | 315 | 415 | ... |
| 1,672 | 1,379 | | 16,095 | 192,634 | 147,925 | 79,447 | 30,200 | 9,502 | 119,149 | 67,775 |

### ISLINGTON AND NORTH-WEST DISTRICT.

Comprising Islington, Holloway, Highbury, Hampstead, Highgate, St. Pancras, Stoke Newington, Tottenham, &c.

| No. of Beds. | No. of Beds Daily Occupied. | HOSPITALS. | In-patients. | Out-patients. | Total Expenditure. | Income. Charitable. | Proprietary. | Patients' Payments | Total Income. | Legacies not included in previous column. |
|---|---|---|---|---|---|---|---|---|---|---|
| | | | | | £ | £ | £ | £ | £ | £ |
| 107 | 98 | Great Northern Central ... ... | 1,421 | 22,385 | 9,624 | 7,146 | 794 | 422 | 8,362 | 2,925 |
| 30 | 18 | Hampstead Hospital... ... ... | 270 | 744 | 2,756 | 753 | 952 | 461 | 2,166 | 200 |
| 120 | 67 | London Temperance ... ... ... | 1,066 | 14,668 | 9,356 | 5,785 | 1,569 | 240 | 7,594 | 3,379 |
| 53 | 50 | North-West London... ... ... | 683 | 21,227 | 4,347 | 3,304 | 139 | 36 | 3,479 | ... |
| 210 | 184 | University College ... ... ... | 3,151 | 44,201 | 19,226 | 8,571 | 4,016 | 28 | 12,615 | 2,716 |
| 60 | 54 | North London Consumption ... | 434 | 3,265 | 5,906 | 6,014 | 117 | 38 | 6,169 | 105 |
| 170 | 18 | London Fever... ... ... | 692 | ... | 15,112 | 7,610 | 1,948 | 2,290 | 11,848 | 323 |
| 28 | 16 | Invalid Asylum ... ... ... | 191 | ... | 867 | 630 | 122 | 150 | 902 | 50 |
| 16 | 15 | Children's Home Hospital, Barnet... | 73 | ... | 483 | 375 | 72 | 180 | 627 | ... |
| 10 | 8 | Enfield Cottage ... ... ... | 90 | ... | 616 | 416 | 11 | 79 | 506 | ... |
| 26 | 11 | St. Saviour's, for Cancer ... ... | 80 | ... | 1,696 | 965 | ... | 456 | 1,421 | 25 |
| 830 | 539 | | 8,151 | 106,490 | 59,989 | 41,569 | 9,740 | 4,380 | 55,689 | 9,723 |
| | | DISPENSARIES. | | | | | | | | |
| ... | ... | Camden Provident ... ... ... | ... | 962 | 284 | 24 | ... | 233 | 257 | ... |
| ... | ... | Hampstead Provident ... ... | ... | 11,182 | 1,073 | 302 | 56 | 688 | 1,046 | ... |
| ... | ... | Holloway and North Islington ... | ... | 3,838 | 936 | 344 | 57 | 346 | 747 | 50 |
| ... | ... | Islington ... ... ... ... | ... | 11,085 | 873 | 317 | 16 | 552 | 885 | 50 |
| ... | ... | St. Pancras and Northern ... ... | ... | 1,722 | 559 | 285 | 90 | 86 | 461 | 300 |
| ... | ... | Stamford Hill, &c. ... ... ... | ... | 6,158 | 716 | 511 | 144 | ... | 655 | 500 |
| 830 | 539 | | 8,151 | 141,437 | 74,430 | 43,352 | 10,103 | 6,285 | 59,740 | 10,623 |

### WESTMINSTER DISTRICT.

Comprising Westminster City and Liberties.

| No. of Beds. | No. of Beds Daily Occupied. | HOSPITALS. | In-patients. | Out-patients. | Total Expenditure. | Charitable. | Proprietary. | Patients' Payments | Total Income. | Legacies not included in previous column. |
|---|---|---|---|---|---|---|---|---|---|---|
| | | | | | £ | £ | £ | £ | £ | £ |
| 175 | 148 | Charing Cross... ... ... ... | 2,117 | 24,949 | 14,673 | 7,753 | 2,391 | 143 | 10,287 | 2,481 |
| 220 | 183 | King's College ... ... ... | 2,539 | 26,175 | 20,307 | 10,239 | 1,868 | 134 | 12,241 | 4,406 |
| 205 | 128 | Westminster ... ... ... ... | 2,142 | 18,437 | 17,604 | 10,757 | 3,137 | ... | 13,894 | 3,819 |
| 134 | 116 | Ventnor, for Consumption ... ... | 778 | ... | 11,160 | 5,414 | 1,810 | 3,285 | 10,509 | 4,623 |
| 18 | 12 | Grosvenor, for Women and Children | 135 | 2,379 | 1,659 | 1,086 | 5 | 344 | 1,435 | 500 |
| 60 | 46 | Hospital for Women ... ... | 661 | 5,110 | 6,019 | 3,491 | 116 | 469 | 4,076 | 1,272 |
| 25 | 19 | National, for Diseases of Heart, &c. | 167 | 1,669 | 2,324 | 1,616 | 26 | 307 | 1,949 | 727 |
| 30 | 21 | Royal Westminster Ophthalmic ... | 451 | 9,777 | 2,242 | 2,164 | 370 | 73 | 2,607 | 50 |
| 50 | 49 | Royal Orthopædic ... ... ... | 134 | 704 | 2,013 | 1,199 | 490 | 124 | 1,813 | ... |
| 26 | 21 | Hospital for Diseases of the Throat | 548 | 8,268 | 3,724 | 916 | 246 | 2,801 | 3,963 | 350 |
| 9 | 7 | Royal Ear ... ... ... ... | 213 | 2,130 | 746 | 162 | ... | 595 | 757 | ... |
| ... | ... | Dental ... ... ... ... ... | ... | 49,860 | 2,019 | 1,653 | 186 | 239 | 2,078 | 150 |
| 20 | 15 | Gordon, for Fistula ... ... ... | 232 | 784 | 1,327 | 491 | ... | 827 | 1,318 | ... |
| 26 | 19 | St. Peter's, for Stone ... ... | 448 | 4,637 | 3,938 | 833 | 537 | 2,186 | 3,556 | ... |
| 998 | 784 | | 10,565 | 154,879 | 89,755 | 47,774 | 11,182 | 11,527 | 70,483 | 18,378 |
| | | DISPENSARIES. | | | | | | | | |
| ... | ... | Public ... ... ... ... ... | ... | 3,196 | 754 | 413 | 145 | ... | 558 | ... |
| ... | ... | St. George and St. James ... ... | ... | 3,764 | 616 | 398 | 11 | 21 | 430 | ... |
| ... | ... | St. George, Hanover Square ... | ... | 1,244 | 578 | 401 | 10 | 181 | 592 | ... |
| ... | ... | Western ... ... ... ... | ... | 8,774 | 1,475 | 438 | 106 | 595 | 1,439 | 450 |
| ... | ... | Westminster General ... ... | ... | 6,505 | 582 | 348 | 145 | 68 | 561 | 122 |
| 998 | 784 | | 10,565 | 178,362 | 93,760 | 49,772 | 11,899 | 12,392 | 74,063 | 18,950 |

### STRATFORD AND EAST-END DISTRICT.

Comprising Bethnal Green, Tower Hamlets, West Ham, Whitechapel, Hackney, Stepney, Limehouse, Poplar, and the East.

| No. of Beds. | No. of Beds Daily Occupied. | HOSPITALS. | In-patients. | Out-patients. | Total Expenditure. | Charitable. | Proprietary. | Patients' Payments | Total Income. | Legacies not included in previous column. |
|---|---|---|---|---|---|---|---|---|---|---|
| | | | | | £ | £ | £ | £ | £ | £ |
| 125 | 95 | German ... ... ... ... | 1,226 | 19,251 | 9,766 | 9,475 | 2,338 | 365 | 12,178 | 1,060 |
| 776 | 617 | London... ... ... ... ... | 9,954 | 184,617 | 72,131 | 21,997 | 24,383 | 489 | 46,869 | 15,052 |
| 64 | 53 | Poplar ... ... ... ... ... | 1,007 | 16,624 | 5,287 | 6,584 | 446 | ... | 7,030 | 100 |
| 57 | 27 | West Ham, &c. ... ... ... | 417 | 20,223 | 4,024 | 4,848 | 270 | ... | 5,118 | 500 |
| 164 | 102 | City of London for Dis. of the Chest | 918 | 16,063 | 11,961 | 6,595 | 257 | ... | 6,852 | 9,395 |
| 102 | 60 | East London, for Children ... ... | 1,238 | 34,104 | 6,477 | 6,811 | 875 | ... | 7,686 | 1,166 |
| 92 | 41 | Mrs. Gladstone's Home ... ... | 705 | ... | 1,180 | 582 | 380 | ... | 962 | 200 |
| 13 | 8 | East End Mothers' Home ... ... | 230 | 259 | 1,789 | 1,024 | 349 | 47 | 1,420 | 100 |
| 1,393. | 1,003 | | 15,695 | 291,141 | 112,615 | 57,916 | 29,298 | 901 | 88,115 | 27,573 |
| | | DISPENSARIES. | | | | | | | | |
| ... | ... | Eastern ... ... ... ... | ... | 6,395 | 756 | 311 | 340 | 60 | 711 | ... |
| ... | ... | Hackney Provident ... ... ... | ... | 362 | 237 | 32 | ... | 215 | 247 | ... |
| ... | ... | London... ... ... ... ... | ... | 2,157 | 463 | 170 | 269 | ... | 439 | ... |
| ... | ... | Queen Adelaide's ... ... ... | ... | 7,597 | 503 | 401 | 203 | 48 | 652 | 9 |
| ... | ... | Tower Hamlets ... ... ... | ... | 4,504 | 759 | 704 | 27 | 172 | 903 | ... |
| ... | ... | Whitechapel Provident ... ... | ... | 5,043 | 707 | 33 | ... | 644 | 677 | ... |
| 1,393 | 1,003 | | 15,695 | 317,199 | 116,040 | 59,567 | 30,137 | 2,040 | 91,744 | 27,582 |

### A SUMMARY OF THE WORK DONE IN 1895.

**It will be seen from the following summary that the Voluntary Hospitals and Medical Charities of London, during the twelve months ending 31st December, 1895, relieved over one million four hundred and sixty-three thousand patients at a cost of £718,264. The Ordinary Income only amounted to £576,447, leaving a deficiency of £141,817 on the year's work. The Legacies received in 1895 amounted to £176,672, being £39,538 in excess of the amount received in 1894.**

| No. of Beds. | No. of Beds Daily Occupied. | Hospitals and Dispensaries. | In-patients. | Out-patients. | Total Expenditure. | Income. | | | Total Income. | Legacies not included in previous column. |
|---|---|---|---|---|---|---|---|---|---|---|
| | | | | | | Charitable. | Proprietary. | Patients' Payments. | | |
| | | | | | £ | £ | £ | £ | £ | £ |
| 1,638 | 1,095 | Newington and South District ... | 15,125 | 192,284 | 104,780 | 43,649 | 37,778 | 13,960 | 95,387 | 2,399 |
| 662 | 432 | City and East Central District ... | 7,292 | 158,526 | 56,858 | 31,164 | 8,454 | 5,523 | 45,141 | 11,880 |
| 998 | 784 | Westminster District ... ... | 10,565 | 178,362 | 93,760 | 49,772 | 11,899 | 12,392 | 74,063 | 18,950 |
| 1,412 | 1,119 | St. Marylebone and West Central District ... ... ... ... | 11,513 | 199,056 | 124,471 | 56,226 | 25,572 | 9,425 | 91,223 | 37,463 |
| 1,672 | 1,379 | Kensington and West District ... | 16,095 | 192,634 | 147,925 | 79,447 | 30,200 | 9,502 | 119,149 | 67,775 |
| 830 | 539 | Islington and North-West District | 8,151 | 141,437 | 74,430 | 43,352 | 10,103 | 6,285 | 59,740 | 10,623 |
| 1,393 | 1,003 | Stratford and East-End District... | 15,695 | 317,199 | 116,040 | 59,567 | 30,137 | 2,040 | 91,744 | 27,582 |
| 9,005 | 6,351 | | 88,436 | 1,379,498 | 718,264 | 363,177 | 154,143 | 59,127 | 576,447 | 176,672 |

### THE NEED AND VALUE OF PERSONAL DEVOTION.

WHEN the question is put, "What do the personal devotion and enthusiasm of hospital workers mean to the sick and the methods applied for the alleviation of sickness and suffering in London to-day?" we may truthfully reply, "It means everything."

Without the sustained devotion and enthusiasm of the workers the stupendous task set them in the maintenance of the hospitals would be an impossible one, and not all the memories of the beneficent part these institutions have played in the social history of the nation, nor the prestige of their great traditions and far-reaching usefulness, would save them from collapse.

And if the enthusiasm and devotion of the workers, the ability to be inspired rather than depressed by difficulty and discouragement, are necessary to the maintenance of the work, they are not less indispensable to its embellishment. Let us present the case as we may, the sordid details of hospital finance will never fascinate anybody; to not a few their intrusion seems revolting; something whose presence, like poverty of any sort, is to be resented.

The pleasures of perversity are enduring, and of all potent factors in philanthropy, what can compare with caprice? In the chances of life, rich institutions, like rich people, are always surest of prizes, and of all possible depositories of gifts and legacies who so likely as those who have enough and to spare?

The hospitals have been slow to turn this knowledge to account. They have displayed their indigence, and it has proved unattractive. Let them now exhibit their wealth. What stores they have! Its evidences meet you at every turn, wealth of love and sympathy, of tenderness and self-sacrifice and devotion to duty, which make a hospital ward fruitful of living lessons.

Here, at least, there is nothing of the poverty told of in the appeal, and so heartily despised. If it exist at all, it is artistically concealed. The doctors scarcely suspect it, for their costliest prescriptions are seldom questioned. The patients know nothing of it, for all their needs are supplied, while the requirements and even the luxuries of life are not denied them.

Those who would learn all the nation owes to the hospitals must come and see for themselves what manner of work is done and what manner of workers they are who do it. The love of duty unselfishly undertaken and carried through at any cost, which makes heroes in all callings, can nowhere be more commonly found than in the wards of our hospitals; and if one would read all the story aright, one must look for it in the hearts of the sufferers.

That is what the clergy who are to preach on Hospital Sunday might do with advantage. Let them catch some of the spirit of the workers, then we might hope for preachers who would eschew the trite tale of poverty, and present the case in its splendour, setting forth boldly the power and riches of the hospitals; for how can they be beggars who have in their keeping the treasure of a people's health, or poor to whom all the nation is debtor?

### A HINT TO THE WISE.

WE desire to direct the special attention of our readers to the advertisement pages of this Hospital Sunday Supplement, which are confined entirely to statements concerning individual institutions. Editorially, we are necessarily compelled to deal mainly with the general question of the adequate maintenance of our voluntary hospitals, their paramount necessity for Londoners, and the claims which they have individually and collectively upon everyone, whether a permanent or temporary resident in the metropolis of the empire. We have carefully gone through these advertisements, and are struck with the convincing eloquence displayed by the secretaries who have written the appeals they contain. Apart, however, from the appeal element, all, or nearly all, describe tersely the objects of each institution, the volume of the work done, and its special needs at the moment. We can promise any reader who will attentively peruse these advertisements that he will be well rewarded for his trouble. May he further be led to follow the example of more than one wealthy philanthropist last year by taking steps to permanently preserve this Special Supplement for reference when making his gifts to hospitals and kindred charities. We have not space to give a subscription form this year, but in these days of enlightenment no one needs such a reminder, nor is it necessary to identify each gift with this Special Supplement.

## Figures and Tables

**Figure f1:**
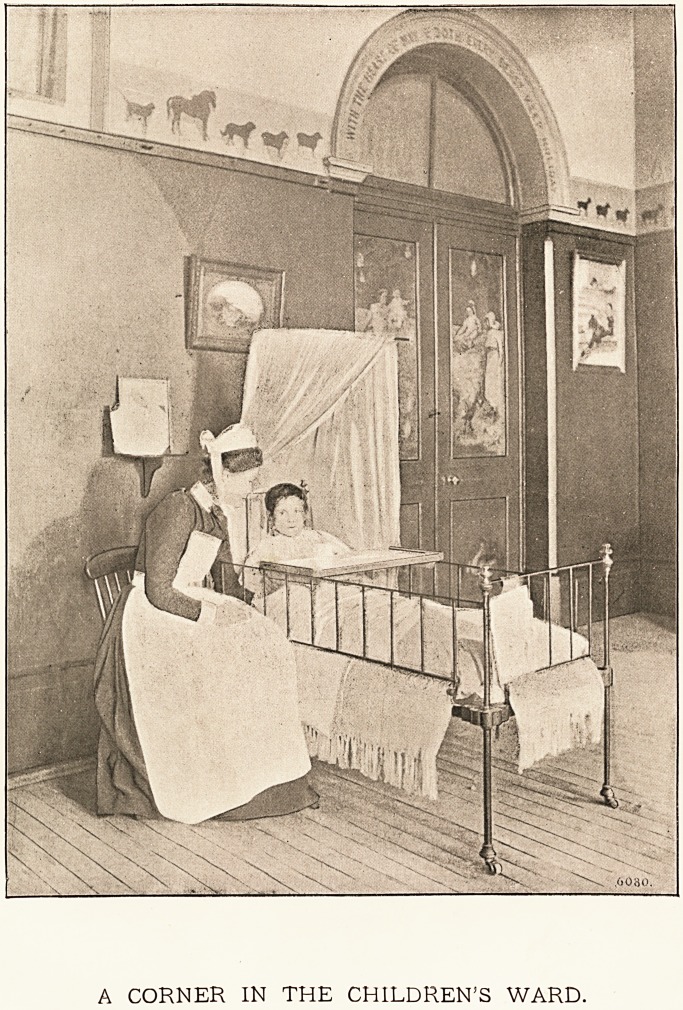


**Figure f2:**
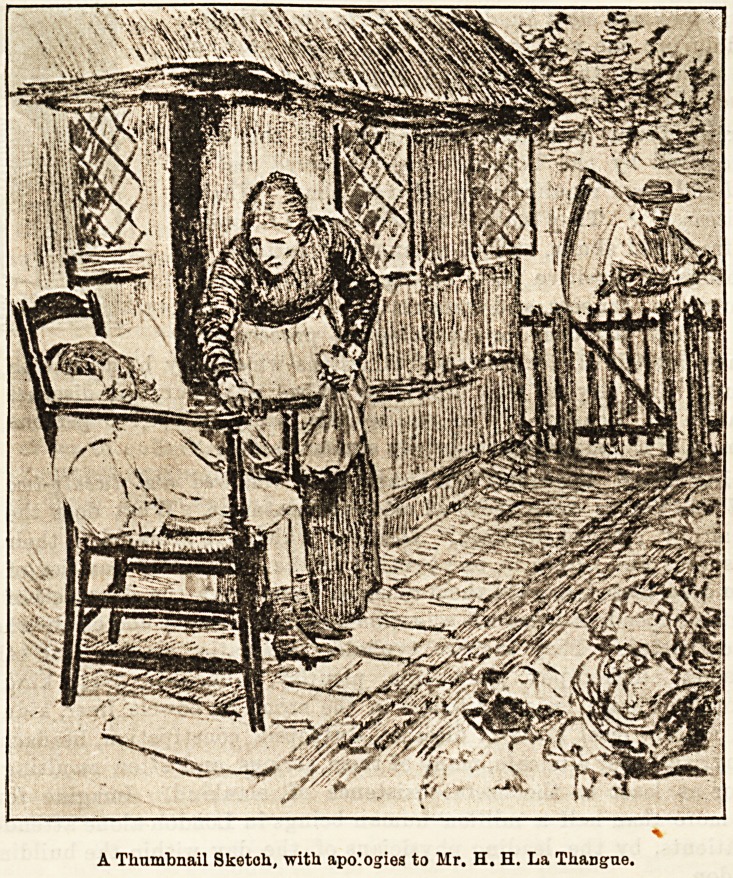


**Figure f3:**
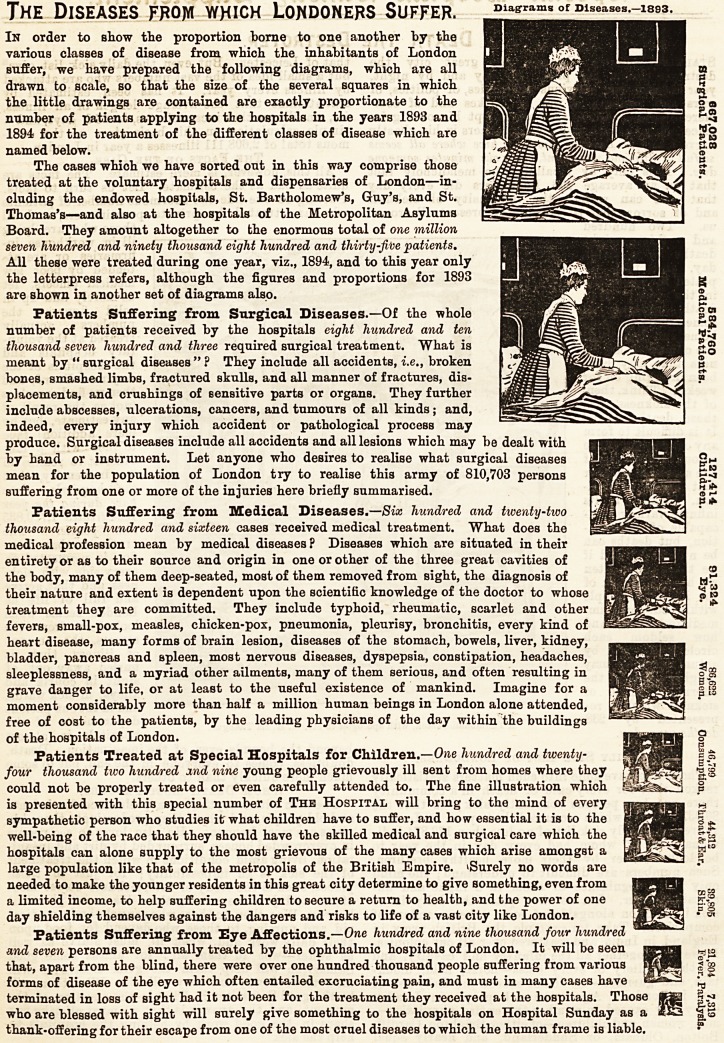


**Figure f4:**
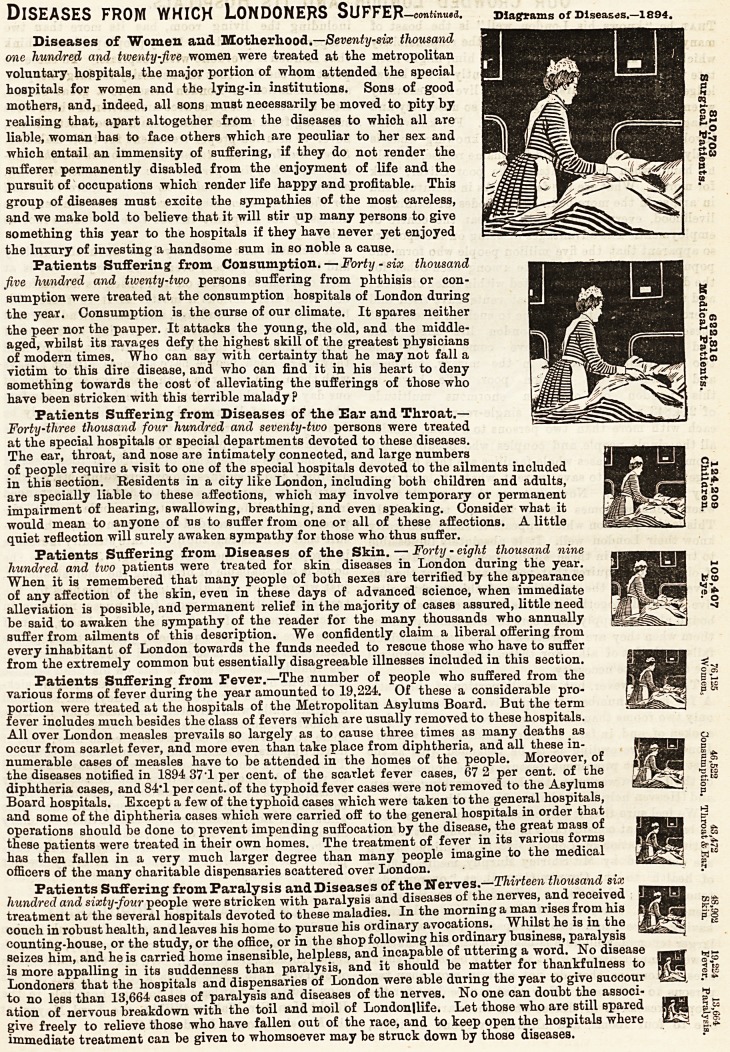


**Figure f5:**
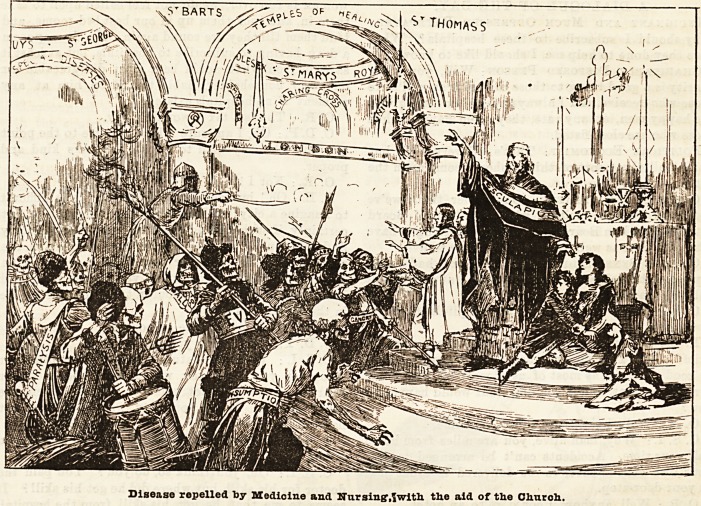


**Figure f6:**
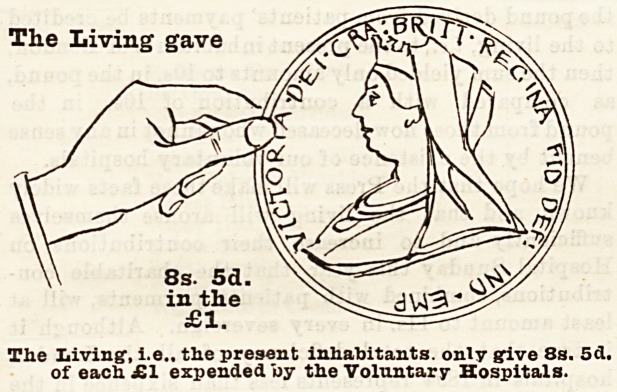


**Figure f7:**
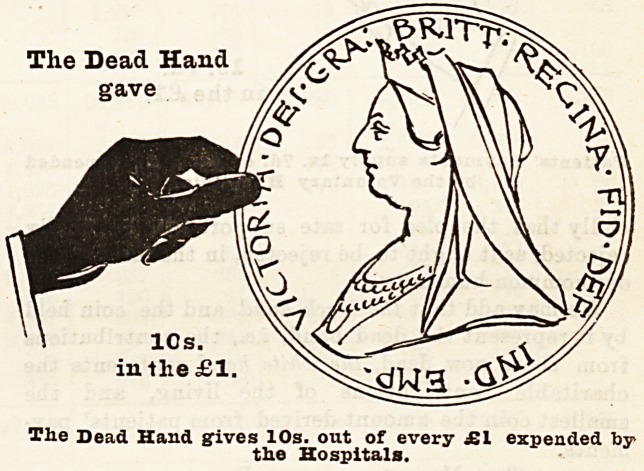


**Figure f8:**